# Ultrasound-Derived Diaphragm Contractile Reserve as a Marker of Clinical Status in Patients With Cystic Fibrosis

**DOI:** 10.3389/fphys.2021.808770

**Published:** 2022-01-10

**Authors:** Fanny Gabrysz-Forget, Anne-Catherine Maynard-Paquette, Aileen Kharat, François Tremblay, Maité Silviet-Carricart, Annick Lavoie, Martin Girard, Bruno-Pierre Dubé

**Affiliations:** ^1^Département de Médecine, Centre Hospitalier de l’Université de Montréal (CHUM), Montréal, QC, Canada; ^2^Département de Médecine, Service de Pneumologie, Centre Hospitalier de l’Université de Montréal (CHUM), Montréal, QC, Canada; ^3^Département d’Anesthésiologie, Centre Hospitalier de l’Université de Montréal (CHUM), Montréal, QC, Canada; ^4^Centre de Recherche du Centre Hospitalier de l’Université de Montréal (CRCHUM), Carrefour de l’Innovation et de l’Évaluation en Santé, Montréal, QC, Canada

**Keywords:** diaphragm, cystic fibrosis, ultrasound, lung function, respiratory physiology, respiratory muscle assessment

## Abstract

**Introduction:** In patients with cystic fibrosis (CF), the monitoring of respiratory muscle activity using electromyography can provide information on the demand-to-capacity ratio of the respiratory system and act as a clinical marker of disease activity, but this technique is not adapted to routine clinical care. Ultrasonography of the diaphragm could provide an alternative, simpler and more widely available alternative allowing the real-time assessment of the diaphragm contractile reserve (DCR), but its relationship with recognized markers of disease severity and clinical outcomes are currently unknown.

**Methods:** Stable patients with CF were prospectively recruited. Diaphragm ultrasound was performed and compared to forced expiratory volume in 1 s (FEV_1_), residual volume (RV), handgrip strength, fat-free mass index (FFMI), serum vitamin levels, dyspnea levels and rate of acute exacerbation (AE). Diaphragm activity was reported as DCR (the ratio of tidal-to-maximal thickening fractions, representing the remaining diaphragm contractility available after tidal inspiration) and TFmax (representing maximal diaphragm contractile strength). Inter-observer reliability of the measurement of DCR was evaluated using intra-class correlation analysis.

**Results:** 110 patients were included [61 males, median (interquartile range), age 31 (27–38) years, FEV_1_ 66 (46–82)% predicted]. DCR was significantly correlated to FEV_1_ (rho = 0.46, *p* < 0.001), RV (rho = −0.46, *p* < 0.001), FFMI (rho = 0.41, *p* < 0.001), and handgrip strength (rho = 0.22, *p* = 0.02), but TFmax was not. In a multiple linear regression analysis, both RV and FFMI were independent predictors of DCR. DCR, but not TFmax, was statistically lower in patients with > 2 exacerbations/year (56 ± 25 vs. 71 ± 17%, *p* = 0.001) and significantly lower with higher dyspnea levels. A ROC analysis showed that DCR performed better than FEV_1_ (mean difference in AUROC 0.09, *p* = 0.04), RV (mean difference in AUROC 0.11, *p* = 0.03), and TFmax at identifying patients with an mMRC score > 2. Inter-observer reliability of DCR was high (ICC = 0.89, 95% CI 0.84–0.92, *p* < 0.001).

**Conclusion:** In patients with CF, DCR is a reliable and non-invasive marker of disease severity that is related to respiratory and extra-pulmonary manifestations of the disease and to clinical outcomes. Future studies investigating the use of DCR as a longitudinal marker of disease progression, response to interventions or target for therapy would further validate its translation into clinical practice.

## Introduction

The evaluation of diaphragm function using ultrasonography is a simple, rapid, reproducible, and non-invasive surrogate marker of diaphragm contractile capacity, which is most frequently reported using maximal diaphragm thickening fraction (TFmax) (Houston et al., 1995; Ueki et al., 1995; Gottesman and McCool, 1997; Boussuges et al., 2009, 2020; Boon et al., 2014; Goligher et al., 2015; Tuinman et al., 2020). In the intensive care unit (ICU) setting, loss of diaphragm muscle strength is both frequent and multifactorial, and ultrasound-confirmed diaphragm weakness is associated with weaning outcome, length of mechanical ventilation and survival (Maher et al., 1995; Vassilakopoulos et al., 1998; Jiang et al., 2004; Hermans et al., 2010; Kim et al., 2010, 2011; Demoule et al., 2013; Supinski and Callahan, 2013; DiNino et al., 2014; Ferrari et al., 2014).

The recent recognition that diaphragm ultrasound can also provide additional, more dynamic information on the state of the respiratory system than the measurement of TFmax alone, has renewed interest for this technique outside of the ICU. In particular, the assessment of diaphragm contractile reserve (DCR, defined as the ratio of tidal-to-maximal diaphragm thickening fractions) can provide an instantaneous estimation of the demand-to-capacity balance of the respiratory system, which conceptually relates to the neural drive to breathe. Recently, studies have described the clinical relevance of DCR in patients with chronic obstructive respiratory disease (COPD) by demonstrating its significant relationships to clinical symptoms and lung function in stable patients. In patients undergoing a COPD exacerbation, DCR also correlated to hospital stay, ventilatory failure and readmission (Numis et al., 2014; Alqahtani et al., 2020; Maynard-Paquette et al., 2020; Rittayamai et al., 2020).

In patients with cystic fibrosis (CF), respiratory muscle activity monitoring using electromyography (EMG) of the parasternal muscles has been shown to be sensitive to clinical deterioration during an episode of exacerbation (Reilly et al., 2012), suggesting that respiratory muscle activity in CF patients can act as a marker of the state of the respiratory system and be responsive to clinical evolution. The ultrasonographic measurement of DCR could provide a simple, widely available and non-invasive alternative method to similarly assess the demand/capacity ratio of the respiratory system in CF patients, which in turn could provide clinicians with a novel tool allowing the evaluation of respiratory status in this population. However, data on the use of DCR in CF patients are currently lacking. In a previous study by Enright et al. (2007), the presence of lower fat-free mass (FFM) was associated with lower diaphragm contractile strength. However, the absolute contractile strength values of patients with low FFM remained within the expected normal values for healthy subjects (Boon et al., 2013), therefore limiting its clinical usefulness. This study did not include a dynamic marker of the demand-to-capacity ratio of the diaphragm such as DCR. In addition, the relationship between DCR and common markers of disease severity in CF such as peripheral muscle strength loss, vitamin deficiency and airway obstruction remain unclear. In light of this, the present study was designed with the following objectives: (1) to identify the putative determinants and predictors of DCR among demographic, physiological and nutritional characteristics of CF patients, and (2) to compare the performance of DCR and TFmax as markers of relevant clinical outcomes (dyspnea levels and exacerbation frequency) in this population.

## Materials and Methods

### Study Participants

Patients with CF were prospectively recruited at the Centre Hospitalier de l’Université de Montréal (CHUM) during their routine follow-up appointments at the CF clinic between January 2019 and October 2020.

Inclusion criteria were: a definitive diagnosis of CF based on the CF Foundation diagnostic criteria (Farrell et al., 2017), age > 17 years and clinical stability [defined as the presence of all of the following: absence of hospitalization for any cause in the last 4 weeks, absence of acute infectious exacerbation requiring oral or intravenous antibiotic treatment in the last 4 weeks, forced expiratory volume in 1 s (FEV_1_) value at the time of inclusion within 5% of a patient’s recent stable values and confirmation by the attending physicians of a clinical state representing the patient’s baseline status].

Exclusion criteria were: any known neurological or rheumatological degenerative disease, any suspicion of diaphragm paresis or phrenic palsy (evaluated using medical records and chest radiographs), active cancer and any other local factors that could interfere with the performance or results of diaphragm ultrasound (thoracic wound, chest tubes, past lung resection surgery, pleural effusion).

Informed written consent was obtained from all participants. The study was approved from the ethics board of the CHUM (CE 16.082) and was conducted in compliance with the Declaration of Helsinki.

### Data Collection

Demographic data including age, gender, height, weight, body mass index (BMI), CF genotype, and associated medical comorbidities were collected from medical files. Current microbiological colonizing organisms and serum nutritional markers [serum transthyretin, vitamin A, E, and 25(OH)D levels] were measured at inclusion. The number of acute exacerbation episodes requiring intravenous antibiotic therapy (either during hospitalization or as outpatient treatment) in the year before inclusion in the study was retrieved from medical files and the local CF database. Complete pulmonary function tests were performed according to American Thoracic Society/European Respiratory Society guidelines (Macintyre et al., 2005; Miller et al., 2005; Wanger et al., 2005). All subjects were asked to grade their dyspnea levels on the mMRC scale (Maher et al., 1995).

### Body Composition and Peripheral Muscle Strength

Body composition was assessed using a bioelectrical impedance scale (BC-554 body composition monitor, Tanita Corporation of America, Illinois, United States). Total body weight, percent body fat, muscle mass and fat free mass were recorded. Fat-free mass index (FFMI) was calculated as fat-free mass/(height squared).

Peripheral muscle strength was assessed using a handgrip dynamometer. Patients were asked to perform maximal isometric contraction of the hand and forearm muscles against the dynamometer. The mean value of three trials [with a 15-s rest between trials, allowing a rapid evaluation while maintaining high inter-trial repeatability (Trossman, 1989)] was reported for each hand. For analyses purposes, handgrip strength was reported as the sum of left and right values.

### Diaphragm Ultrasound

Ultrasound assessment of diaphragm thickness and thickening was performed using a linear 4–12 MHz probe (Sparq, Phillips Healthcare, United States), as previously described (Wait et al., 1989; Bruin et al., 1997; Gottesman and McCool, 1997; Boussuges et al., 2009; Boon et al., 2013; Matamis et al., 2013; Baria et al., 2014; Maynard-Paquette et al., 2020; Rittayamai et al., 2020). Briefly, measurements were performed with patients lying down, with the head of the bed raised at an angle of approximately 30 degrees. The ultrasound probe was placed perpendicular to the chest wall in a sagittal oblique plane at the midaxillary line between the 9th and 10th intercostal spaces (i.e., at the level of the zone of apposition). The diaphragm was identified as a three-layered structure comprising two hyperechoic lines, respectively, representing the pleural and peritoneal membranes, and a middle hypoechoic layer representing the diaphragmatic muscle fibers. Diaphragm thickness was measured in B-mode using electronic calipers in three distinct conditions: at the end of tidal expiration (TDE), at the end of tidal inspiration (TDItidal), and at the end of a maximal inspiratory maneuver (TDImax). For each measurement, at least three breathing cycles were recorded, and their average value was reported. Measurements were performed on both the right and left hemidiaphragm. In the case where maximal inspiration resulted in significant lung sliding that displaced the region of maximal thickness outside of the ultrasound window, TDE, TDitidal, and TDImax were instead measured at a lower intercostal level (usually the 11th intercostal space) to allow the measurement of all variables in the same acoustic window (Supplementary Figure 1).

The thickening fraction of the diaphragm during tidal breathing (TFtidal), representing the magnitude of diaphragm activation required for tidal breathing, was calculated as 100*[(TDItidal–TDE)/TDE]. The thickening fraction of the diaphragm during maximal inspiratory effort (TFmax) representing maximal diaphragm activation capacity, was calculated as 100*[(TDImax – TDE)/TDE]. Diaphragm contractile reserve (DCR), representing the remaining diaphragm capacity available after tidal inspiration, was calculated as 100-(TFtidal/TFmax). Diaphragm weakness was defined by the presence of a TFmax value < 20%. Ultrasound measurements were performed by three members of the study team with experience in diaphragm ultrasound (FGF, ACMP, and AK). To evaluate inter-observer reliability, all frozen ultrasound images were anonymized and remeasured, in random order, by an experienced observer from the study team not having participated in the initial data collection (BPD).

### Statistical Analyses

Normality of the data was evaluated using the Kolmogorov-Smirnov test, and data was reported as mean (standard deviation), median (interquartile range), or n (percent), where appropriate.

To identify the putative determinants and predictors of DCR, correlations or differences between diaphragm ultrasound variables and demographic, anthropometric, nutritional/body composition and lung function variables were evaluated using Spearman correlations or chi-square tests, where appropriate, and partial correlation analyses were used to control for potential confounders [such as FEV_1_ in the correlation between DCR and residual volume (RV)]. A multiple regression model aiming at identifying independent predictors of DCR was computed using variables having shown statistically significant correlations in univariate analyses.

To compare the performances of DCR and TFmax as markers of clinical outcomes, differences in ultrasound variables according to dyspnea levels on the mMRC scale and exacerbation rate (with 2 exacerbations in the year preceding inclusion as pre-specified cut-off point) were evaluated using one-way ANOVA and independent *t*-tests, where appropriate. Receiver operating characteristics (ROC) curves were constructed to evaluate and compare the predictive ability of DCR, TFmax, FEV_1_, and RV in the evaluation of dyspnea. Statistical comparison of the area under the ROC curves (AUROC) were performed using the Hanley-McNeil test (Hanley and McNeil, 1983).

Inter-observer reliability for the calculation of DCR and TFmax were reported graphically using Bland-Altman plots and quantified using intra-class correlation coefficients (two-way mixed model for absolute agreement) (Bland and Altman, 1986).

As left and right-sided diaphragm values of DCR were found to be highly correlated (see section “Results”), values from the right-sided measurements were used for analyses. This is representative of clinical practice, as right-sided diaphragm ultrasound is usually easier and faster to perform because of the improved acoustic window provided by the underlying liver tissue (Matamis et al., 2013).

Statistical significance was defined as *p* < 0.05. All analyses were performed using SPSS version 25 (IBM corporation, Armonk, NY, United States) and MedCalc, version 19.4 (MedCalc Software, Ostend, Belgium).

## Results

Clinical characteristics of the study population are presented in Table 1. A total of 110 patients were recruited (61 males), of which 40 had a homozygous ΔF508 genotype. Mean FEV_1_ was moderately reduced (65 ± 24% predicted) and severely reduced in 11% of patients.

**TABLE 1:** *Patients characteristics*.

N	110
Age, *years*	31 [27–38]
Males, *n*	61 (55)
Homozygous ΔF508 genotype, *n*	40 (36)
BMI, *kg/m^2^*	22 [20–24]
Fat-free mass, *kg*	42 [36–48]
Fat-free mass index, *kg/m^2^*	15 [14–17]
**Comorbidities**	
Diabetes	57 (52)
Pancreatic insufficiency	93 (85)
Osteoporosis	16 (15)
**Bacterial colonization**	
*Pseudomonas* sp., *n*	83 (76)
*Staph. aureus, n*	50 (46)
*Meth-resistant staph aureus, n*	19 (17)
*B. cepacia, n*	15 (14)
*Stenotrophomonas* sp*., n*	31 (28)
*Mycobacteria* sp*., n*	18 (16)
*Aspergillus fumigatus, n*	24 (22)
1-year exacerbations	1 [0–2]
Dyspnea, *mMRC scale*	1 [1–2]
**Laboratory studies**	
Glycated hemoglobin, %	5.6 [5.3–6.2]
Vitamin A, *mmol/L*	1.55 (0.48)
25-(OH)-D, *nmol/L*	82 (22)
Vitamin E, *mmol/L*	23.9 [19.3–30.4]
Zinc, *mmol/L*	12.5 (2.5)
Transthyretin, *mg/L*	211 (59)
**Pulmonary function testing**	
FEV_1_/FVC ratio, %	69 (14)
FEV_1_, %*predicted*	65 (24)
FEV_1_ > 70% predicted, *n*	48 (44)
FEV_1_ 35–70% predicted, *n*	49 (45)
FEV_1_ < 35% predicted, *n*	13 (11)
Inspiratory capacity, %*predicted*	95 (24)
FRC, %*predicted*	117 (24)
RV, %*predicted*	170 (54)
TLC, %*predicted*	106 (12)
DLCO, %*predicted*	94 (24)

*Data presented as n (%), mean [standard deviation] or median [interquartile range]. BMI, body mass index; mMRC, modified medical research council; FEV_1_, forced expiratory volume in 1 s; FVC, forced vital capacity; FRC, functional residual capacity; RV, residual volume; TLC, total lung capacity; DLCO, diffusion capacity of the lung for carbon monoxide.*

### Diaphragm Ultrasound

Table 2 displays the values for all ultrasound variables, which were similar between the right- and left-hemidiaphragms. Correlations between the right and left-sided values of DCR were statistically significant (rho = 0.88, *p* < 0.001, respectively). Only one patient (0.9%) had overt diaphragm weakness (mean of right and left TFmax values of 19%).

**TABLE 2:** Distribution of diaphragm ultrasound variables.

	Right hemidiaphragm	Left hemidiaphragm
	Median (IQR)	Median (IQR)
End-expiratory thickness (TDE), *mm*	1.97 [1.66–2.36]	1.88 [1.30–2.20]
End-inspiratory thickness (TDItidal), *mm*	2.50 [2.14–3.04]	2.45 [2.07–2.90]
End-inspiratory thickness (TDImax), *mm*	3.80 [2.94–4.80]	3.77 [2.91–4.81]
Tidal thickening fraction (TFtidal), %	21 [14–36]	26 [17–44]
Maximal thickening fraction (TFmax), %	86 [54–126]	89 [61–133]
Diaphragm contractile reserve (DCR)	70 [50–85]	67 [53–83]

*IQR, interquartile range.*

### Determinants and Predictors of Diaphragm Contractile Reserve in the Study Population

There were no statistically significant correlations between DCR and age, vitamin A, E, D, and zinc levels (all *p* > 0.05). However, DCR was significantly correlated to transthyretin levels (rho = 0.27, *p* = 0.05), FFMI (rho = 0.41, *p* < 0.001), and handgrip strength (rho = 0.22, *p* = 0.02). There were no statistical differences in DCR values between groups based on sex, comorbidities, or bacterial colonization status (all *p* > 0.05).

Table 3 displays the correlation coefficients observed between both DCR and TFmax and the main lung function testing results. There were statistically significant relationships between DCR and FEV_1_ and RV. In contrast, TFmax was not significantly correlated to either forced expiratory flows, lung volumes or diffusing capacity. After correction for FEV_1_, the relationship between DCR and RV remained statistically significant (*p* = 0.016).

**TABLE 3:** Correlations between ultrasound variables and lung function testing.

	TFmax		DCR	
	Correlation (rho)	*p*-value	Correlation (rho)	*p*-value
FEV_1_, %*predicted*	0.11	0.25	0.46	<0.001
RV, %*predicted*	–0.08	0.44	–0.46	<0.001
DLCO, %*predicted*	0.14	0.14	0.29	0.002

*TFmax, maximal thickening fraction of the diaphragm; DCR, diaphragm contractile reserve; FEV1, forced expiratory volume in 1 s; FVC, forced vital capacity; FRC, functional residual capacity; RV, residual volume; TLC, total lung capacity; DLCO, diffusion capacity of the lung for carbon monoxide.*

To identify independent predictors of DCR, a multiple linear regression models was performed using the following variables: handgrip strength, FFMI, FEV_1_ (percent predicted) and RV (percent predicted). Both RV and FFMI were independently and significantly related to DCR value (Table 4). None of these variables was independently related to TFmax, although FEV_1_ and FFMI showed relationships at the limit of statistical significance. In univariate analysis, there was a weak, but statistically significant relationship between FFM and TFmax (rho = 0.25, *p* = 0.01). However, substituting FFM for FFMI in the multiple regression analysis did not show a statistically significant relationship (*p* = 0.09).

**TABLE 4:** Multiple linear regression models assessing the independence of the relationship between clinical predictors and ultrasound results.

	DCR		TFmax	
	Beta coefficient	*p*-value	Beta coefficient	*p*-value
Handgrip strength, *N*	0.06	0.48	0.21	0.05
FFMI, *kg/m^2^*	0.32	<0.001	0.21	0.05
FEV_1_, *percent predicted*	0.24	0.052	0.11	0.44
RV, *percent predicted*	–0.25	0.03	0.006	0.97

*FEV1, forced expiratory volume in 1 s; RV, residual volume.*

### Relationships Between Diaphragm Ultrasound Variables and Clinical Outcomes

**FIGURE 1:**
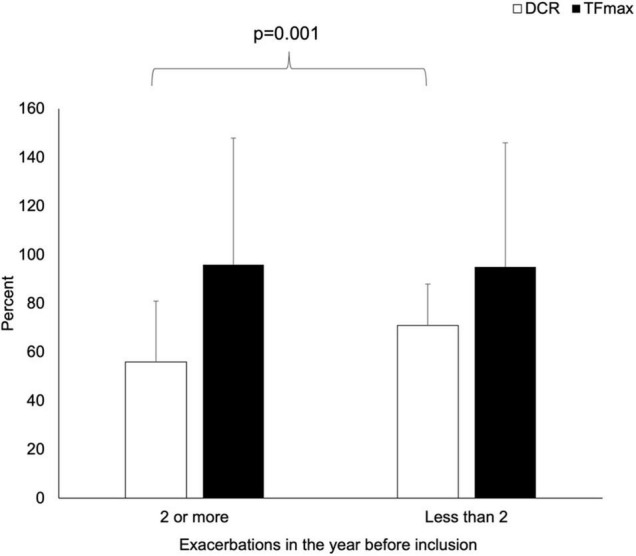
Comparison of the values of TFmax and DCR among CF patients according to acute exacerbation frequency in the year before enrollment.

Compared with patients with fewer exacerbations, those with two or more exacerbations in the year before enrollment had lower values of DCR (56 ± 25 vs. 71 ± 17%, *p* = 0.001) but similar values of TFmax (96 ± 52 vs. 95 ± 51%, *p* = 0.96) (see Figure 1).

**FIGURE 2:**
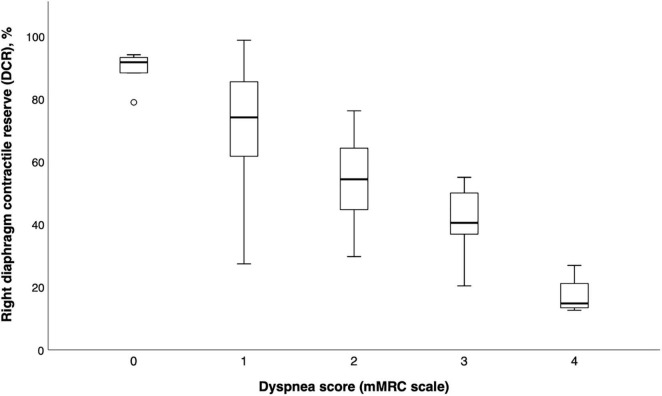
Boxplot graph of diaphragm contractile reserve values according to dyspnea levels (mMRC scale).

Similarly, DCR was significantly lower in patients with greater dyspnea scores on the mMRC scale (one-way ANOVA *F* = 22.5, *p* < 0.001) (see Figure 2).

**FIGURE 3:**
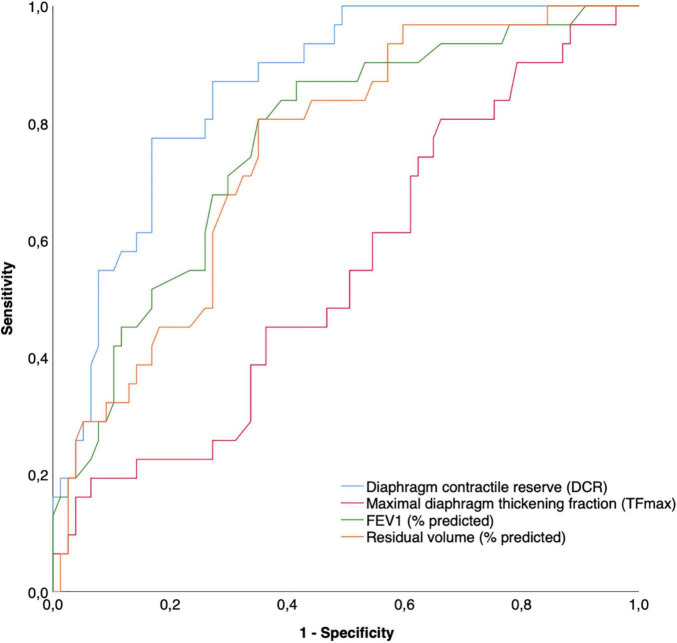
Receiver operating characteristics curves comparing different ultrasound and physiological variables in the prediction of the presence of a higher (>2 on mMRC scale) dyspnea level.

A ROC analysis aiming at comparing the sensitivity and specificity of TFmax, DCR, FEV_1_ (percent predicted) and RV (percent predicted) at identifying patients with an mMRC score > 2 showed that DCR, FEV_1_, and RV all had statistically significant predictive values, while TFmax did not (Table 5). Among these, DCR performed better than FEV_1_ (mean difference in AUROC 0.09, *p* = 0.04) and RV (mean difference in AUROC 0.11, *p* = 0.03) (see Figure 3).

**TABLE 5:** ROC analysis comparing ultrasonographic and lung function variables in the identification of patients with higher dyspnea scores (mMRC > 2).

	AUROC	95% confidence interval	*p*-value
Maximal diaphragm thickening fraction (TFmax)	0.55	0.43–0.67	0.44
Diaphragm contractile reserve (DCR)	0.86	0.79–0.93	<0.001
FEV_1_ (Percent predicted)	0.77	0.67–0.86	<0.001
RV (Percent predicted)	0.75	0.65–0.84	<0.001

*AUROC, area under the receiver operating characteristic curve; FEV1, forced expiratory volume in 1 s; RV, residual volume.*

### Inter-Observer Reliability

Bland-Altman plots evaluating the inter-observer reliability for the measurement of DCR and TFmax are presented in Supplementary Figures 2, 3. The agreement between observers remained broadly in the limits of agreement across the spectrum of observed DCR and TFmax values. Intra-class correlations for the measurements of DCR and TFmax were high (0.89, 95% CI 0.84–0.92, *p* < 0.001 and 0.98, 95% CI 0.97–0.99, *p* < 0.001, respectively).

## Discussion

The main results of our study can be summarized as follows: in our cohort of CF patients with varying degrees of disease severity and airflow obstruction, (1) DCR was significantly and independently related to markers of disease severity that encompass both the respiratory and metabolic involvement in CF (i.e., FEV_1_, RV, and FFMI) and (2) DCR was significantly related to clinically relevant outcomes such as exacerbation rate and dyspnea levels (even outperforming FEV_1_ for this outcome). Taken together, these results suggest that a dynamic measurement of diaphragm contractile activity, such as DCR, can provide clinicians with a novel, non-invasive and widely available tool to estimate respiratory disease burden in patients. These findings are paving the way for studies investigating the use of DCR as a longitudinal marker of disease progression, response to interventions, and as a guide for therapeutic management.

Our finding that DCR is significantly related to the severity of airflow obstruction and lung hyperinflation is not unexpected. Indeed, the progressive airway obstruction, hyperinflation and abnormalities in respiratory mechanics characterizing CF are well described factors leading to an increase in tidal respiratory workload and respiratory drive to breathe, which eventually translate in a higher tidal-to-maximal contractile action from the respiratory muscles (Jolley et al., 2009; Murphy et al., 2011; Reilly et al., 2011, 2012). The fact that a clear relationship between physiological markers of airway obstruction and ultrasound-derived diaphragm activity was identified validates the clinical relevance of using DCR to dynamically capture changes in diaphragm activation. As the mean values of TFmax observed in our cohort remained well within the normal range of values expected for normal subjects (Boon et al., 2013), the observed decline in DCR with worsening airflow obstruction was likely due to an increase in tidal thickening fraction relative to its maximal thickening fraction, rather than a decrease in maximal contractile capacity. This echoes the EMG findings of Reilly et al. (2011, 2012) and further supports the promising ability of DCR to adequately represent the increase in neural to breathe associated with the increased expiratory flow limitation observed in CF.

Our finding that DCR, but interestingly not TFmax, was significantly and independently related to FFMI warrants attention. In a previous study by Enright et al. (2007) maximal diaphragm contractility was compared between CF patients with low and preserved FFM using the thickening ratio of the diaphragm (TR—a marker closely related to the TFmax used in our study). Patients with low FFM had lower TR values than those with preserved FFM and healthy controls, possibly indicating a relationship between peripheral and respiratory muscle weakness (Enright et al., 2007). In contrast, although we did observe a weak correlation between TFmax and FFM in univariate analysis, this relationship did not persist when controlled for handgrip strength, FEV_1_ and RV. This raises doubts on the independence of the relationship between body composition and intrinsic diaphragm contractile strength in the CF population.

In our results, the dynamic marker of the demand-to-capacity ratio of the respiratory system (DCR), was significantly related to FFMI even after controlling for FEV_1_ and RV. Although this finding does not allow to draw conclusions on the causative mechanisms of this relationship, it suggests that factors other than airway obstruction and lung hyperinflation are playing a role in the increased respiratory workload and drive to breathe of patients with low FFMI.

In patients with low FFMI (either due to malnutrition, COPD, or restrictive lung disease), factors such as musculo-skeletal alterations to the ribcage mechanics, inefficient alveolar ventilation, higher oxygen cost of breathing during exercise and psychological factors such as anxiety are putative mechanisms by which respiratory drive (and therefore DCR) could be heightened (Sridhar et al., 1994; Creutzberg et al., 1998; Budweiser et al., 2008; Yohannes et al., 2018). Future research investigating the relationship between peripheral and respiratory muscle composition and integrity in the CF population could target these mechanisms.

From the clinical viewpoint, the most relevant finding of our study is the significant relationships observered between DCR and important markers of disease severity, including exacerbation rate and dyspnea. These findings support the notion that DCR can be conceptualized as an instant marker of the demand-to-capacity ratio of the diaphragm, and therefore dynamically represent the clinical status of the respiratory system. These results also complement recent findings of Rittayamai et al. (2020) reporting a significantly lower DCR in COPD patients with increased disease severity and frequent exacerbations. Although there is little doubt that FEV_1_ will remain the standard in evaluating overall disease burden in CF for the foreseeable future (Kerem et al., 2014; Szczesniak et al., 2017; Keogh et al., 2019; Liou et al., 2020), our results support the validity of DCR as a potentially new marker of disease severity that could complement FEV_1_ in the longitudinal monitoring of CF patients and their response to therapeutic interventions. In addition, our results raise the possibility that improving DCR with tailored therapeutic interventions such as inspiratory muscle training (IMT) could positively impact dyspnea levels and clinical outcomes, although this remains to be demonstrated in prospective clinical trials.

The main strength of the present study are that we included a large sample of patients with a wide spectrum of disease severity, while concomitantly investigating both respiratory and extra-pulmonary markers of disease severity. It also showed that highly repeatable ultrasonographic measurements can be obtained by multiple operators assessing diaphragm activity. However, some limitations also need to be acknowledged. First, we did not compare our ultrasound measurements of DCR to a more standardized mean of evaluating neural drive to breathe such as the electrical activity of the diaphragm (measured using an esophageal catheter or EMG). Although these techniques would have provided a more formal comparison of the potential of DCR to act as a marker of muscular activity, they are cumbersome to use in clinical practice as they are time-consuming, require highly specialized equipment and are relatively invasive for patients. An EMG monitoring of the parasternal muscles (rather than of the diaphragm itself) could be a less invasive method to correlate ultrasonographic measurement such as DCR to neural drive to breathe in future studies, especially given the data supporting the validity of parasternal EMG as a surrogate of diaphragm EMG in the CF population (Reilly et al., 2011). Independently of the correlation that can exist between EMG and ultrasound variables, our results provide evidence that DCR correlates to meaningful clinical variables providing grounds from which clinicians and researchers can further validate its clinical usefulness.

Secondly, we limited our evaluation to clinically stable CF patients. This allows the comparison of DCR to stable-state values of respiratory function testing and body composition, however, limiting the extent to which the study findings can be generalized to unstable patients. Undoubtedly, further research is required before DCR can fully translate into a reliable clinical tool, in order to validate its ability to adequately assess situations where respiratory drive is dynamically altered, such as during an exacerbation event. Nevertheless, the significant relationships between DCR and clinical outcomes across the spectrum of disease severity found in our study lend credibility to its potential as an responsive, dynamic, clinical tool.

Third, we must acknowledge that the strength of the relationship between TFmax, which we used as a maker of maximal diaphragm activation, and more objective measurements of the pressure-generating capacity of the diaphragm has been challenged in some publications (Tuinman et al., 2020). It is possible that the inherent discrepancy between TFmax and “true” diaphragm contractile capacity may have weakened the relationship between our measurement of TFmax and clinical outcomes. We believe that this further strengthens our conclusion that a dynamic marker of respiratory drive such as DCR could provide more valid and more clinically relevant information to clinicians, especially in a population of patients where overt diaphragm dysfunction is not the main clinical issue.

## Conclusion

We provide the first evidence that DCR, a simple, non-invasive and repeatable ultrasonographic estimation of the current contractile activity of the diaphragm, is significantly related to both respiratory and extra-pulmonary markers of disease severity in patients with CF. In contrast, maximal diaphragm contractility such as TFmax was not. We also show that DCR is associated to adverse clinical outcomes related to disease prognosis, including exacerbation rate and dyspnea levels. These results lay the first basis for the translation of DCR from a research setting to clinical practice. Future studies investigating whether DCR can act as a longitudinal monitoring tool for disease progression, response to treatment or even as a target for therapy would further strengthen its clinical relevance.

## Data Availability Statement

The raw data supporting the conclusions of this article will be made available by the authors, without undue reservation.

## Ethics Statement

The studies involving human participants were reviewed and approved by the Comité d’Éthique de la Recherche du Centre Hospitalier de l’Université de Montréal (CHUM). The patients/participants provided their written informed consent to participate in this study.

## Author Contributions

FG-F, A-CM-P, AK, and B-PD contributed to data collection and writing of the manuscript. FG-F, A-CM-P, AK, AL, MG, and B-PD contributed to data analysis and interpretation. FT, MS-C, and AL contributed to critical revision of the article. All authors approved the final version of the manuscript and contributed to the conception and design of the study.

## Conflict of Interest

The authors declare that the research was conducted in the absence of any commercial or financial relationships that could be construed as a potential conflict of interest.

## Publisher’s Note

All claims expressed in this article are solely those of the authors and do not necessarily represent those of their affiliated organizations, or those of the publisher, the editors and the reviewers. Any product that may be evaluated in this article, or claim that may be made by its manufacturer, is not guaranteed or endorsed by the publisher.
