# Supplementary figures and images for: Ultrasound-Derived Diaphragm Contractile Reserve as a Marker of Clinical Status in Patients With Cystic Fibrosis
Source: Front Physiol. 2022 Jan 10;12:808770. doi: 10.3389/fphys.2021.808770 (PMC8784523; doi:10.3389/fphys.2021.808770)

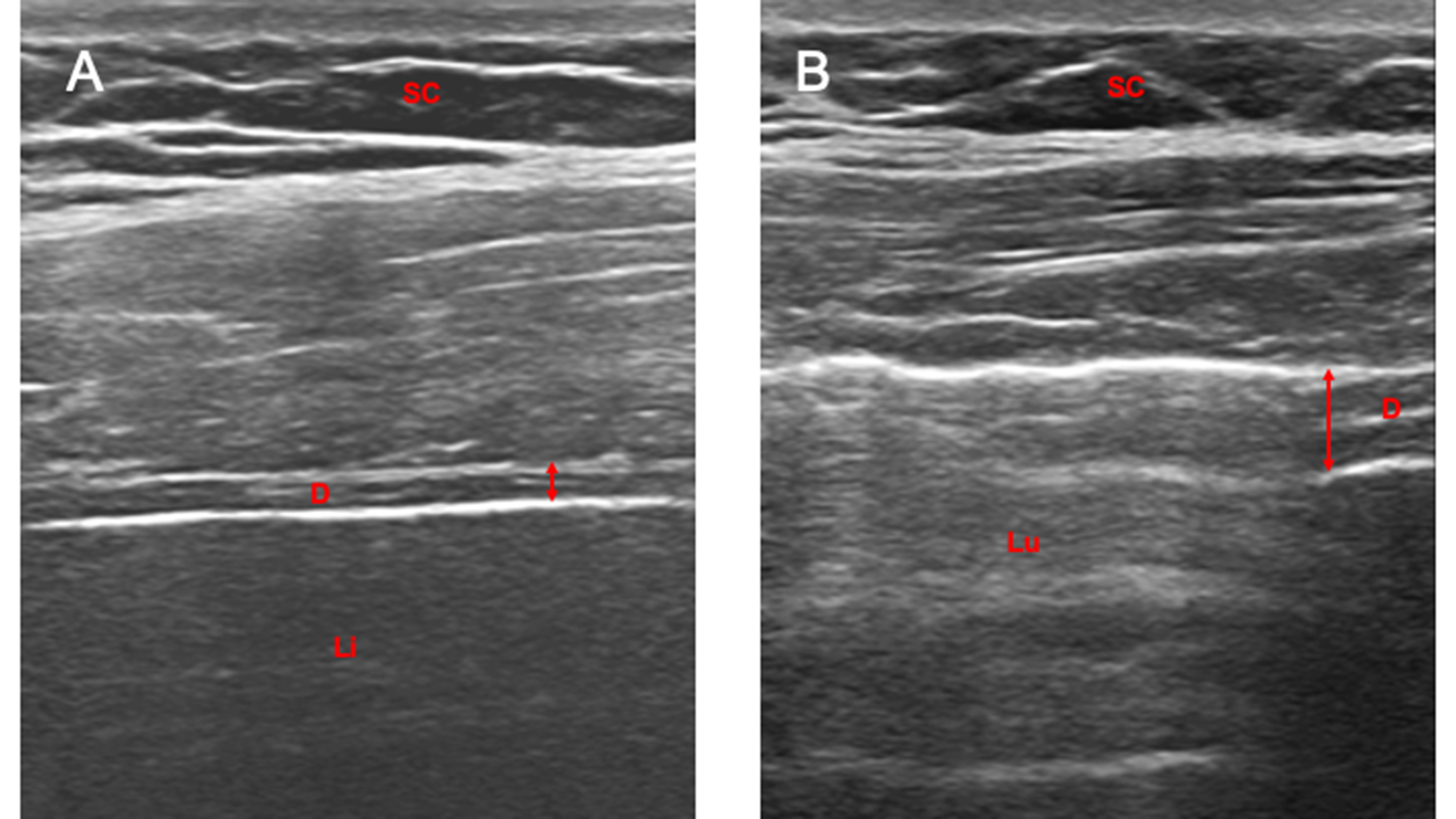

Supplement: Supplementary file 1 [file Image_1.TIFF]

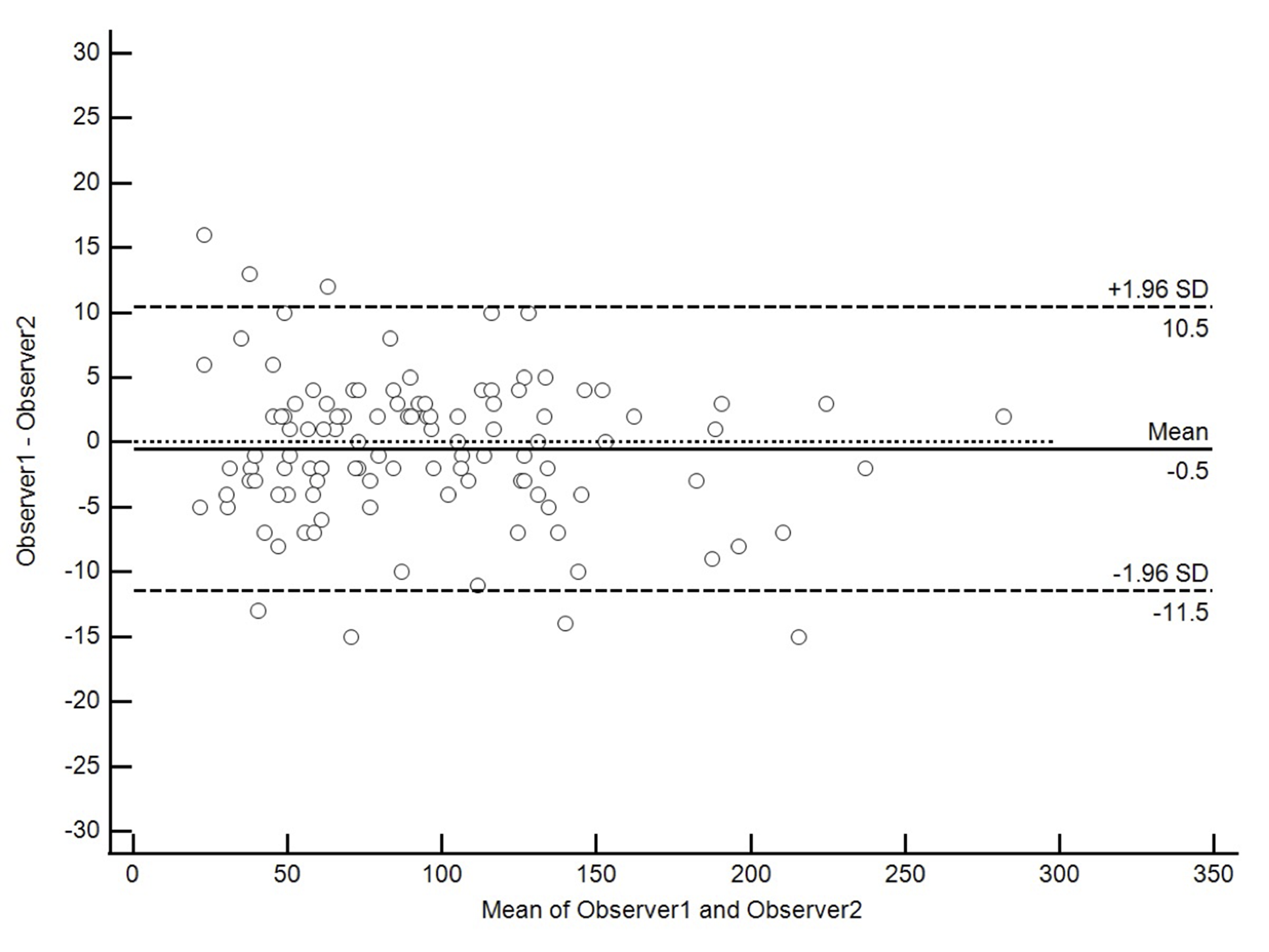

Supplement: Supplementary file 2 [file Image_2.TIFF]

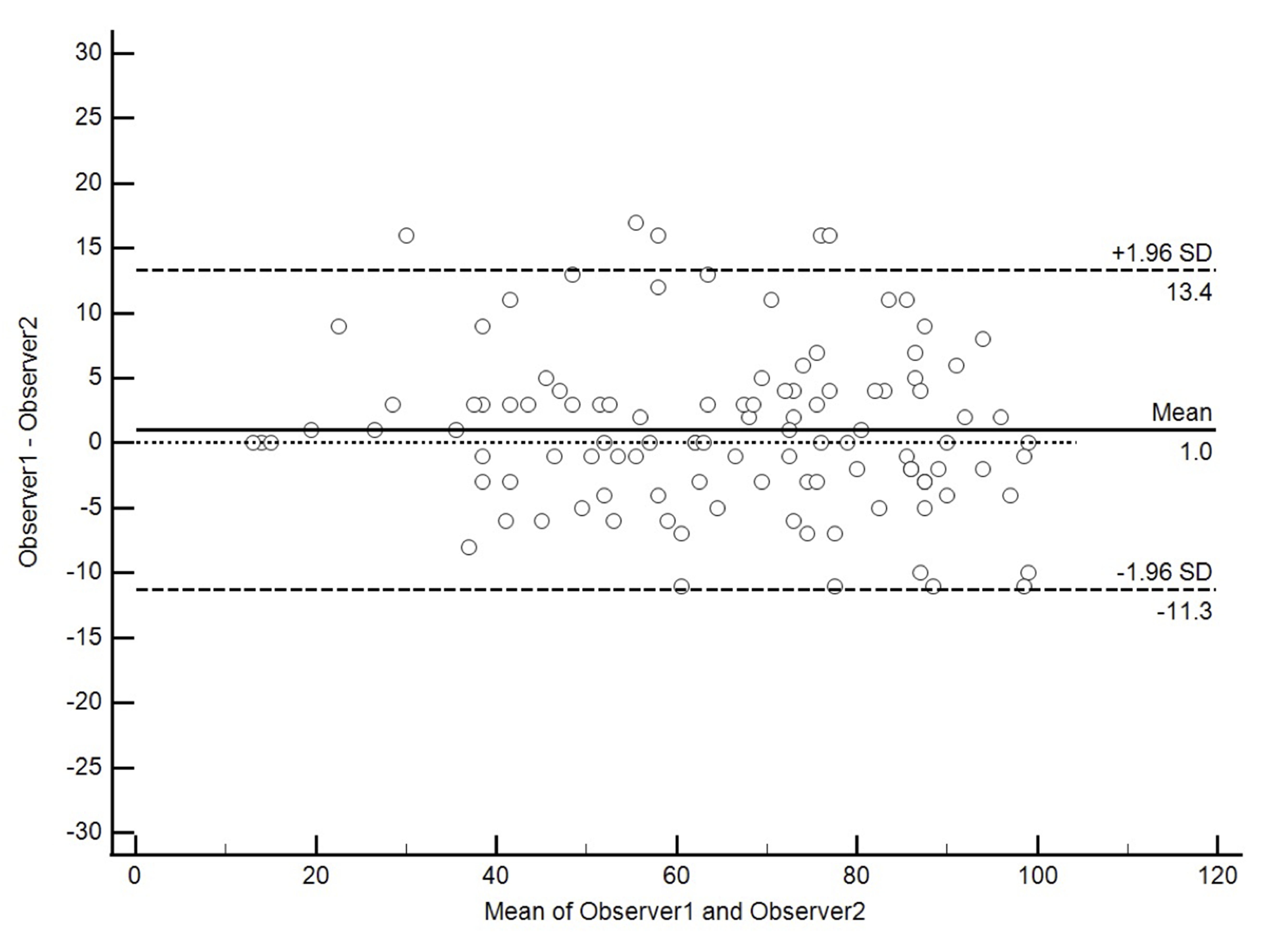

Supplement: Supplementary file 3 [file Image_3.TIFF]
