# Supplementary material for: Ultrasound-Derived Diaphragm Contractile Reserve as a Marker of Clinical Status in Patients With Cystic Fibrosis
Source: Front Physiol. 2022 Jan 10;12:808770. doi: 10.3389/fphys.2021.808770 (PMC8784523; doi:10.3389/fphys.2021.808770)
Supplement: Supplementary file 4 [file Table_1.docx]

**Ultrasound-derived diaphragm contractile reserve as a marker of clinical status in patients with cystic fibrosis**

Fanny Gabrysz-Forget, Anne-Catherine Maynard-Paquette, Aileen Kharat, François Tremblay, Maité Silviet-Carricart, Annick Lavoie, Martin Girard and Bruno-Pierre Dubé

**Supplemental material**

**Methods**

*Inter-observer reliability*

Bland-Altman plots for the evaluation of inter-observer reliability for the measurement of TFmax and DCR are presented in eFigure 1 and 2. The agreement between observers remained broadly in the limits of agreement across the spectrum of observed DCR and TFmax values. Intra-class correlations for the measurements of DCR and TFmax were high (0.89, 95% CI 0.84-0.92, p<0.001 and 0.98, 95% CI 0.97-0.99, p<0.001, respectively).

**e-Figure legends**

**eFigure 1**. Sample images of B-mode diaphragm ultrasound measurements. Panel A: At end-expiration, the diaphragm is identified as the three-layered structure comprising two hyperechoic lines and a central hypoechoic layer with muscular echotexture. The arrow represents the site of measurement of end-expiratory diaphragm thickness. Panel B: At the end of a maximal inspiration, the lung is displaced caudally and the thickness of the diaphragm increases. The arrow represents the measurement of maximal diaphragm thickness. SC: subcutaneous tissue; D: diaphragm; Li: liver; Lu: lung.

**eFigure 2**. Bland-Altman plot for the inter-observer comparison of the measurement of maximal diaphragm thickening fraction (TFmax)

**eFigure 3.** Bland-Altman plot for the inter-observer comparison of the measurement of diaphragm contractile reserve (DCR)
